# Changes in Lower Extremity Passive Range of Motion and Muscle Strength After Selective Percutaneous Myofascial Lengthening and Functional Physiotherapy in Children With Cerebral Palsy

**DOI:** 10.7759/cureus.67325

**Published:** 2024-08-20

**Authors:** Vasileios C Skoutelis, Zacharias Dimitriadis, Anastasios Kanellopoulos, Argirios Dinopoulos, Panayiotis J Papagelopoulos, Vivian Kanellopoulos, Vasileios A Kontogeorgakos

**Affiliations:** 1 Department of Physiotherapy, ATTIKON University General Hospital, Chaidari, GRC; 2 Department of Physiotherapy, University of West Attica, Egaleo, GRC; 3 School of Medicine, National and Kapodistrian University of Athens, Athens, GRC; 4 Department of Physiotherapy, University of Thessaly, Lamia, GRC; 5 Department of Orthopaedics, IASO Children’s Hospital, Maroussi, GRC; 6 Third Department of Paediatrics, ATTIKON University General Hospital, Chaidari, GRC; 7 First Department of Orthopaedic Surgery, ATTIKON University General Hospital, Chaidari, GRC; 8 Graduate Medical Sciences, Boston University, Boston, USA

**Keywords:** selective percutaneous myofascial lengthening, passive range of motion, muscle strength, functional physiotherapy, contracture, cerebral palsy

## Abstract

Background

Children with cerebral palsy (CP) often experience motor and postural disorders, along with spasticity, muscle weakness, muscle-tendon contractures, and decreased joint range of motion (ROM). Muscle-tendon contractures are typically addressed through orthopaedic surgery to improve joint ROM, which can result in further muscle weakness. This study aimed to investigate the impact of selective percutaneous myofascial lengthening (SPML) combined with functional physiotherapy on joint passive ROM and isometric muscle strength in the lower extremities of children with spastic CP.

Methods

A single-group pre- and post-test design was utilised in this study. Twenty-six children aged five to seven years with spastic CP and Gross Motor Function Classification System levels II-IV underwent the SPML procedure and received nine months of postoperative functional strength training physiotherapy. Joint passive ROM and isometric muscle strength were measured using a universal goniometer and a digital hand-held dynamometer, respectively. Paired-sample t-tests were conducted to compare baseline and follow-up measurements.

Results

Significant improvements (p < 0.05) were observed in passive ROM of hip abduction, straight leg raise, popliteal angle, and ankle dorsiflexion, as well as in isometric strength of hip flexors, extensors, abductors and adductors, knee extensors, and ankle dorsiflexors.

Conclusions

The SPML procedure supported by postoperative functional physiotherapy can effectively address fixed contractures by significantly increasing passive joint ROM and muscle strength. Further research with longer-term follow-up measurements is necessary to confirm and expand upon these findings.

## Introduction

Cerebral palsy (CP) is the most common neurodevelopmental disability affecting 1.6 per 1000 live newborns in high-income countries, including Greece [1]. Spastic CP is the most frequent form, accounting for about 80% of cases [2]. Children with spastic CP often undergo muscle lengthening surgery to address the joint range of motion (ROM) lost due to secondarily developed fixed muscle-tendon contracture [2]. Surgical lengthening of the contractured muscle-tendon unit can be done through an open or percutaneous approach, using techniques such as total tenotomy, hemi-tenotomy, intramuscular tenotomy, and myofascial/aponeurotic lengthening. Traditional techniques have been shown to weaken spastic muscles, so recent efforts have focused on finding optimal surgical techniques and rehabilitation protocols to optimise muscle length while preserving strength [3-5].

Selective percutaneous myofascial lengthening (SPML) has been proposed as a method to minimise surgical trauma, decrease postoperative pain, facilitate immediate mobilisation and weight-bearing exercises, and enable same-day hospital discharge [6]. Recent studies indicate that SPML, when combined with postoperative functional therapy, can enhance walking and gross motor function as well as joint active ROM during gait [6-9]. Nevertheless, there is limited information available regarding the impact of SPML on lower extremity muscle strength and joint ROM [9,10].

This study aimed to investigate the effects of SPML surgery combined with functional physiotherapy on joint passive ROM and lower extremity muscle strength in children with spastic CP aged five to seven years old.

## Materials and methods

Study design

This was a single-group, pre- and post-test study conducted alongside a non-randomised controlled trial (non-RCT) protocol, as previously described in detail in another paper [6]. Two examiners from a gait and motion analysis centre collected the measurements at baseline, within four weeks before surgical intervention, and nine months after orthopaedic surgery and physiotherapy. The study protocol was conducted with approval from the Scientific and Ethical Council of the ATTIKON University General Hospital, Chaidari, Attica, Greece (ΕΒΔ 2199/14-03-2017). The study followed the principles of the Declaration of Helsinki and was registered in the Australian New Zealand Clinical Trials Registry (ACTRN12618001535268). Informed consent was obtained from the parents of all participants.

Participants

Children with spastic CP, who were assigned to the intervention group of the non-RCT [6] based on specific inclusion/exclusion criteria were included in this study. A key inclusion criterion was the need for surgical muscle-tendon lengthening due to fixed muscle contractures in the lower extremities, which were determined through physical examination and kinematic/spatiotemporal gait analysis. A key exclusion criterion was the need for concomitant osteotomy as reconstructive surgery for bony deformities. For further details on the inclusion and exclusion criteria, please refer to the non-RCT study [6].

A priori G*Power analysis showed that a minimum sample size of 15 participants would be sufficient to identify a significant effect of intervention within a single group (power = 0.80, alpha = 0.05, effect size = 0.80, two-tailed). The total sample size was set at 26 children based on the number of intervention participants in the non-RCT [6].

Intervention

The combined programme of SPML surgery and nine-month functional physiotherapy has been detailed in previous publications [6,9]. In summary, SPML surgery was performed by a skilled, specialised, and experienced paediatric orthopaedic surgeon. The procedure involved lengthening the medial hamstrings (semitendinosus at the distal level, semimembranosus and gracilis at the middle level), hip adductors, and/or gastrocnemius muscles as necessary. SPML included fascial micro-incisions near the muscle-tendon junction through a small skin hole. Alcohol blocks (obturators and/or femoral nerves) were utilised during SPML surgery for cases of excessive reflex-mediated stiffness during lower extremity active and passive ROM. Physiotherapy began shortly after surgery on an as-tolerated basis, following an intensive functional strength training programme. Specific exercises and activities were tailored to meet each child’s individual needs and functional level. Therapy sessions were held five to six times a week for the first six weeks, then reduced to two to three times a week until the programme was completed. Parents actively participated in the physiotherapy process, providing opportunities for motor activity practice with numerous repetitions.

Assessments

Passive ROM measurements were taken by two experienced paediatric physiotherapists using a Gollehon Extendable Goniometer (model 01135, Lafayette Instrument Co, Lafayette, IN). They followed a standardised protocol with specific bony landmarks as described by Norkin and White [11] for the following four lower extremity joint movements bilaterally: hip abduction with the knees extended (to assess hip adductors and gracilis length); straight leg raising (to assess straight leg raise (SLR) and hamstrings length); popliteal angle (to assess hamstrings length); ankle dorsiflexion (supine, non-weight bearing) with the knee extended (to assess gastrocnemius length), and flexed (to assess soleus length). These ROM measurements were selected because children with CP often have limitations in these movements, which can directly impact their gross motor function [12]. Goniometric measurements in CP, when conducted by two trained and experienced examiners following a standardised protocol, have been shown to be reliably acceptable to highly reliable [13].

Isometric muscle strength measurements were conducted by a trained and experienced paediatric physiotherapist using a hand-held dynamometer MicroFET2® (Hoggan Scientific LLC, Salt Lake City, UT). The standardised protocol described by Darras et al. [14] was followed for assessing seven muscle groups bilaterally: hip flexors, extensors, abductors, and adductors; knee flexors and extensors; and ankle dorsiflexors. The dynamometer had a measurement range of 0-300 pound-force (lbf) and was set to the manufacturer’s default setting of 3.0 lbf (high threshold). Handheld dynamometry is considered a reliable method for measuring muscle strength in children with CP, especially when well-defined standardised protocols are utilised by a trained and experienced examiner [15].

Statistical analysis

All data were analysed using SPSS for Mac, version 26.0 (IBM Corp., Armonk, NY). The difference between the pre- and post-intervention means for variables of passive ROM and muscle strength were examined using paired-sample t-tests. The measurements were obtained from each lower extremity (right, left) and were analysed for both the total of lower extremities (n = 52), as well as after dividing them into less- and more-affected lower extremities, based on a preoperative examination performed by the treating orthopaedic surgeon. Significance levels were set at p < 0.05.

## Results

Participants

A total of 26 children with spastic CP were included in the intervention group of the non-RCT [6]. Of these children, 16 were male with a mean age of 6.15 ± 0.73 years. The distribution of Gross Motor Function Classification System (GMFCS) levels was as follows: II = 6, III = 12, and IV = 8. Additionally, there were 11 children with tetraplegia, 13 with diplegia, and two with hemiplegia. These children were physically examined for passive ROM and isometric strength of the lower extremities.

Passive ROM

The paired-sample t-test revealed statistically significant improvements (p < 0.001) in passive ROM for hip abduction, SLR, popliteal angle, and ankle dorsiflexion with the knee extended after the intervention. These improvements were observed in both the total lower extremities and individually in the less- and most-affected lower extremities. Ankle dorsiflexion passive ROM with the knee flexed increased significantly (p < 0.05) in the total lower extremities, but did not show a statistically significant increase (p > 0.05) in the less- and most-affected lower extremities (Table 1).

**Table 1:** Comparison of ROM measurements before and after the intervention. CI, confidence interval; L/UCL, lower/upper confidence limit; n, number of the lower extremities; MD, mean difference between pre- and post-experimental measurements; ROM, range of motion; SD, standard deviation; SLR, straight leg raising.

Passive ROM measurement (degrees)	Lower extremities	n	Mean ± SD		Mean difference and 95% CI of the difference			
			Pre	Post	MD ± SD	LCL	UCL	P-value
Hip abduction	Totally	52	31.85 ± 10.87	37.69 ± 8.07	5.85 ± 7.76	3.69	8.01	<0.001
	Less affected	26	32.04 ± 10.90	37.50 ± 8.39	5.46 ± 7.48	2.44	8.48	0.001
	More affected	26	31.65 ± 11.06	37.88 ± 7.90	6.23 ± 8.16	2.93	9.53	0.001
SLR test	Totally	52	59.90 ± 12.58	73.65 ± 11.16	13.75 ± 15.62	-18.10	-9.40	<0.001
	Less affected	26	62.12 ± 10.02	73.85 ± 11.16	11.73 ± 14.07	-17.41	-6.05	<0.001
	More affected	26	57.69 ± 14.58	73.46 ± 11.38	15.77 ±17.07	-22.66	-8.88	<0.001
Popliteal angle	Totally	52	42.02 ± 13.87	23.46 ± 11.86	18.56 ± 16.01	14.10	23.01	<0.001
	Less affected	26	40.00 ± 12.49	23.08 ± 11.92	16.92 ± 16.50	10.26	23.59	<0.001
	More affected	26	44.04 ± 15.10	23.85 ± 12.03	20.19 ±15.65	13.87	26.51	<0.001
Ankle dorsiflexion-knee extended	Totally	52	2.25 ± 10.52	10.19 ± 8.28	7.94 ± 7.78	-10.11	-5.78	<0.001
	Less affected	26	3.73 ± 10.09	10.96 ± 8.13	7.23 ± 6.59	-9.89	-4.57	<0.001
	More affected	26	0.77 ± 10.93	9.42 ± 8.52	8.65 ± 8.90	-12.25	-5.06	<0.001
Ankle dorsiflexion-knee flexed	Totally	52	12.71 ± 15.52	17.17 ± 4.80	4.46 ± 14.06	-8.38	-0.55	0.026
	Less affected	26	13.69 ± 14.11	17.88 ± 3.79	4.19 ± 12.58	-9.27	0.89	0.102
	More affected	26	11.73 ± 17.02	16.46 ± 5.62	4.73 ± 15.66	-11.05	1.59	0.136

Isometric muscle strength

The paired-sample t-test indicated statistically significant improvements (p < 0.05) in the isometric strength of hip flexors, extensors, abductors and adductors, knee extensors, and ankle dorsiflexors following the intervention. These improvements were observed for the total lower extremities as well as separately for the less- and most-affected lower extremities. There was a non-significant decreasing trend (p > 0.05) in the isometric strength of the knee flexors (Table 2).

**Table 2:** Comparison of muscle strength measurements before and after the intervention. CI, confidence interval; lbf, pound-force; L/UCL, lower/upper confidence limit; n, number of the lower extremities; MD, mean difference between pre- and post-experimental measurements; ROM, range of motion; SD, standard deviation; SLR, straight leg raising.

Strength (lbf)	Lower extremities	n	Mean ± SD		Mean difference and 95% CI of the difference			
			Pre	Post	MD ± SD	LCL	UCL	P-value
Hip flexors	Totally	52	6.94 ± 8.05	9.89 ±7.67	2.96 ± 5.25	-4.42	-1.49	<0.001
	Less affected	26	7.19 ± 8.40	10.37 ± 7.97	3.18 ± 5.39	-5.36	-1.00	0.006
	More affected	26	6.68 ± 7.84	9.41 ± 7.47	2.73 ± 5.17	-4.82	-0.64	0.012
Hip extensors	Totally	52	9.02 ± 9.68	14.04 ± 10.40	5.03 ± 5.53	-6.57	-3.49	<0.001
	Less affected	26	9.56 ± 10.37	15.14 ± 11.20	5.58 ± 6.13	-8.05	-3.10	<0.001
	More affected	26	8.46 ± 9.12	12.94 ± 9.63	4.48 ± 4.91	-6.46	-2.49	<0.001
Hip abductors	Totally	52	3.88 ± 6.55	5.93 ± 7.38	2.05 ± 4.22	-3.23	-0.87	0.001
	Less affected	26	4.10 ± 6.72	6.16 ± 7.67	2.06 ± 4.42	-3.85	-0.27	0.025
	More affected	26	3.65 ± 6.50	5.69 ± 7.23	2.04 ± 4.09	-3.69	-0.39	0.018
Hip adductors	Totally	52	5.36 ± 7.85	6.78 ± 8.91	1.42 ± 3.28	-2.33	-0.50	0.003
	Less affected	26	5.56 ± 8.19	7.05 ± 9.35	1.49 ± 3.31	-2.83	-0.15	0.031
	More affected	26	5.16 ± 7.65	6.50 ± 8.63	1.34 ± 3.19	-2.64	-0.06	0.041
Knee flexors	Totally	52	4.25 ± 6.62	4.02 ± 6.01	0.23 ±3.15	-0.65	1.10	0.605
	Less affected	26	4.51 ± 7.03	4.34 ± 6.46	0.17 ± 3.52	-1.25	1.59	0.812
	More affected	26	3.98 ± 6.32	3.69 ± 5.64	0.29 ± 2.73	-0.81	1.39	0.594
Knee extensors	Totally	52	7.24 ± 7.76	11.46 ± 7.80	4.22 ± 5.70	-5.81	-2.63	<0.001
	Less affected	26	7.72 ± 8.30	12.04 ± 8.18	4.32 ± 6.15	-6.80	-1.83	0.001
	More affected	26	6.77 ± 7.32	10.89 ± 7.52	4.12 ± 5.26	-6.25	-1.99	0.001
Ankle dorsiflexors	Totally	52	1.19 ± 3.01	4.49 ± 6.03	3.30 ± 4.97	-4.68	-1.92	<0.001
	Less affected	26	1.55 ± 3.72	5.01 ± 6.55	3.46 ± 5.01	-5.49	-1.44	0.002
	More affected	26	0.83 ± 2.10	3.97 ± 5.53	3.14 ± 5.02	-5.17	-1.11	0.004

## Discussion

Passive ROM

In this study, fixed muscle contractures were managed using the SPML procedure. The physical examination indicated that the SPML procedure effectively improved passive ROM in the lower extremity joints. The popliteal angle and SLR, common clinical measures for assessing hamstring contractures [16], showed significant improvements for both the total lower extremities and separately for the less- and more-affected lower extremities. The mean popliteal angle approached the normal angle of 20° (23°), with a postoperative increase of 44%. Additionally, the mean passive range of SLR increased by 23%. The positive impact of the SPML procedure on correcting contractures in the hip adductors and gastrocsoleus was confirmed by significant improvements in the passive ROM of hip abduction and ankle dorsiflexion, with hip abduction increasing by 18% and ankle dorsiflexion by 35% with the knee flexed and over 350% with the knee extended.

Although the SPML procedure is likely the least invasive and least traumatic method of muscle lengthening, as it releases minimal myofascial tissue to achieve the desired lengthening without fully disrupting the muscle-tendon unit [8], it appears to be just as successful as both open and percutaneous techniques described in the literature [3,17]. Several publications have shown that percutaneous lengthening yields comparable results in joint passive ROM to traditional open lengthening.

More precisely, in 2010, El Hage et al. compared percutaneous and open hip adductor tenotomy for the first time in 27 children with CP [18]. They found that the percutaneous approach is just as reliable and effective as the open approach. Significant gains were observed in intraoperative passive hip abduction (measured with a universal goniometer), with no neurovascular complications. In this study, each patient underwent both percutaneous and open approaches, with each side serving as self-control [18].

In a similar study design, Hachache et al. included 31 children with CP, with a mean age of 8.4 years and GMFCS levels I-V [19]. They also concluded that both approaches were effective but noted that percutaneous gracilis tenotomy may not be as safe as open gracilis tenotomy, even when performed correctly by an experienced surgeon. This is mainly due to the increased risk of bleeding [19].

In a comparative study of percutaneous and open hamstring lengthening in 87 children with CP (mean age = 8.4 years, GMFCS levels I-V), Nazareth et al. found that percutaneous hamstring lengthening (n = 22) was as effective as open lengthening (n = 65) in improving popliteal angle passive ROM after an average follow-up of 29.4 months postoperatively [20]. Turhan and Arican also reached similar conclusions when evaluating the popliteal angle in children with spastic CP, aged six to 17 years, who underwent hamstring lengthening through either a percutaneous (n = 12) or open (n = 14) approach [21]. No iatrogenic complications were observed in these studies, indicating the safety of the percutaneous approach [20,21].

Using the same study design as El Hage et al. [18], Mansour et al. performed the SPML procedure followed by an open procedure in the medial hamstrings of 18 children, aged seven to 12 years old, with CP [22]. By emphasizing the orthopaedic surgeon's challenge in identifying and evaluating what should be cut percutaneously, the authors concluded that the SPML procedure was not as anatomically effective and safe as the open procedure [22]. This is because the percutaneous procedure resulted in undesirable extensive muscle injury and less improvement in the intraoperative passive popliteal angle.

Nevertheless, based on most research literature, both the SPML procedure itself [8] and the percutaneous approach in general [3,18,20,21] are considered safe surgical interventions with minimal intraoperative complications. These complications include infection, overlengthening, significant hematomas, nerve damage, or paraesthesia. Orthopaedic surgeons stress that any percutaneous technique carries a risk of injuring adjacent neurovascular and muscle-tendon components [20], as it is challenging to visually direct underlying anatomical structures. However, this risk can be mitigated through the safe and proper performance of the percutaneous technique. In summary, the SPML procedure is a delicate surgical technique that necessitates specialised training, experience, knowledge, and dedication to accurately locate anatomical muscle structures and effectively perform myofascial release surgery.

Isometric muscle strength

In this study, the SPML procedure combined with nine months of functional physiotherapy in children with spastic CP was shown to be beneficial for improving strength in six out of seven lower extremity muscle groups that were examined. This improvement is reflected in the positive results seen in enhancing functional mobility [6,7], as indicated by the significant correlation and effect of muscle strength on ambulatory ability and gross motor function [23]. The strength of the hamstrings as knee flexors remained almost unchanged, with a non-significant decrease of 5.4%. However, hamstring strength may have been preserved and even enhanced nine months postoperatively if their action as hip extensors is considered [3].

In the present study, it cannot be definitively stated whether the 55.7% increase in hip extensor strength had a direct impact on hamstring strength, as the hip extension strength assessment was conducted in a knee flexed position that isolates the gluteus maximus and minimises hamstring activation. The SPML of hip adductors (i.e., adductor longus, gracilis), combined with obturator nerve alcohol block, did not negatively affect strength. Isometric hip adductor strength actually increased significantly by 26.5% following the intervention. According to the literature, nerve block with alcohol does not typically impact voluntary muscle strength, as there is a compensatory increase in the recruitment of motor units, which may result in an immediate increase in motor function or even the generation of active movements that were not present before the alcohol block [24].

Furthermore, the significant increase in lower extremity joint passive ROM obtained after SPML of the spastic agonist muscles (hamstrings, hip adductors, gastrocnemius) allowed for an increase in the excursion length of the antagonist muscles and, consequently, exerted a greater antagonist force [25]. This was demonstrated by a significant increase in the isometric strength in knee extensors, hip abductors, and ankle dorsiflexors. Chang et al. found a significant increase in overall muscle strength in 25 children, aged four to 12 years old, with bilateral spastic CP (GMFCS levels II-IV) at six months after lower extremity multilevel myofascial release and ROM/strengthening physiotherapy [26].

With the exception of two studies reporting no significant changes in lower extremity isometric strength of children with CP within nine months or up to two years after open lengthening [27,28], two other studies from the same institute have shown a significant loss of isometric strength in almost all tested muscle groups at 12 months after open lengthening (as part of multilevel surgery) and a six-week intensive physical therapy strengthening programme [3,5]. In fact, the hamstrings remained about 50% weaker in both studies [3,5], but only 27% weaker when lengthened percutaneously [3]. The last two studies [3,5] highlighted the need for further improvement in surgical muscle lengthening techniques and postoperative physiotherapy programmes to preserve and enhance muscle strength and gross motor function.

According to the findings of the present study, the SPML procedure combined with functional physiotherapy appears to successfully meet the demands of modern orthopaedic surgery. This approach aligns with optimizing the patient's functional strength and independence, rather than solely focusing on correcting musculoskeletal deformities. Unlike conventional open or percutaneous lengthening techniques, the SPML procedure resulted in a significant improvement in muscle strength after nine months, rather than a decline or maintenance of it. This improvement may be attributed to the minimally invasive nature of the SPML procedure, allowing for same-day mobilisation and full weight-bearing functional exercises.

The minimal or no loss of strength in the operated muscles could possibly be attributed to the technical execution of the SPML. This procedure mimics the mechanism of a mesh skin graft by creating parallel mini-incisions in the fascia over the muscle surface through a very small skin hole [6,8]. Another possible explanation could be the implementation of functional strength training therapy. The study data support the significant role of strengthening in postoperative physiotherapy protocols, as advocated, acknowledged, and mandated by the current literature evidence on CP.

Clinical implications

The SPML procedure, despite being minimally invasive, successfully achieves the primary goal of orthopaedic surgery: correcting muscle contractures by effectively increasing muscle flexibility, as evidenced by a significant increase in joint passive ROM. Additionally, the SPML procedure seems to minimise or even eliminate the loss of muscle strength, which is a major deterrent side effect of surgically lengthening weak, contractured, spastic muscles in children with CP. This minimally invasive and less traumatic approach enables early and intensive functional physiotherapy to immediately strengthen muscles and enhance mobility.

Limitations

The primary methodological limitation of the study was the absence of a control group, which resulted from insufficient funding to cover the cost of the gait laboratory physical examination. There is abundant evidence supporting the age-related progressive loss of muscle strength and decline in lower-extremity joint passive ROM over time in children with spastic CP who receive conventional rehabilitation therapies without orthopaedic or neurosurgical interventions. It is well-documented that children with spastic CP generally suffer from an underlying primary muscle weakness, which in conjunction with spasticity, inactivity, and physical growth leads to secondary fixed muscle-tendon contractures. This further decreases muscle strength and limits joint ROM before the age of five years. The well-recognised stability or deterioration in gross motor function at around seven years of age in children with CP is strongly associated with the amount of secondary fixed contractures [2,14,23]. This evidence validates the study’s design decision.

Another potential limitation was the failure to measure plantar flexor strength as part of the standardised physical examination protocol. This omission prevented a more in-depth analysis of the impact of the SPML procedure on gastrocnemius strength. However, the gait laboratory working group argues that this is not a significant limitation, as handheld dynamometer measurements of ankle plantar flexors are known to be unreliable [14].

## Conclusions

The findings of this study revealed that the SPML procedure combined with a nine-month functional physiotherapy programme can effectively address fixed muscle contractures and muscle weakness in children with spastic CP. This is achieved by significantly increasing joint passive ROM and isometric muscle strength in the lower extremities. Further research with longer-term follow-up measurements is needed to build upon these findings.
